# Differences in proxy-reported and patient-reported outcomes: assessing health and functional status among medicare beneficiaries

**DOI:** 10.1186/s12874-015-0053-7

**Published:** 2015-08-12

**Authors:** Minghui Li, Ilene Harris, Z. Kevin Lu

**Affiliations:** Department of Clinical Pharmacy and Outcomes Sciences, South Carolina College of Pharmacy, University of South Carolina, 715 Sumter Street, Columbia, SC USA; Department of Pharmaceutical Health Services Research, School of Pharmacy, University of Maryland, 220 Arch Street, Baltimore, MD USA; IMPAQ International, LLC, 10420 Little Patuxent Pkwy, Columbia, MD USA

**Keywords:** Patient-reported outcomes, Proxy-reported outcomes, Health and functional status, Medicare, Elderly, Disabled

## Abstract

**Background:**

Proxy responses are very common when surveys are conducted among the elderly or disabled population. Outcomes reported by proxy may be systematically different from those obtained from patients directly. The objective of the study is to examine the presence, direction, and magnitude of possible differences between proxy-reported and patient-reported outcomes in health and functional status measures among Medicare beneficiaries.

**Methods:**

This study is a pooled cross-sectional study of a nationally representative sample of community-dwelling Medicare beneficiaries from 2006 to 2011. Survey respondents can respond to the Medicare Current Beneficiary Survey either by themselves or via proxies. Health and functional status was assessed across five domains: physical, affective, cognitive, social, and sensory status. Propensity score matching was used to get matched pairs of patient-reports and proxy-reports.

**Results:**

After applying the propensity score matching, the study identified 7,780 person-years of patient-reports paired with 7,780 person-years of proxy-reports. Except for the sensory limitation, differences between proxy-reported and patient-reported outcomes were present in physical, affective, cognitive, and social limitations. Compared to patient-reports, a question regarding survey respondents’ difficulties in managing money was associated with the largest proxy response bias (relative risk, RR = 3.83). With few exceptions, the presence, direction, and magnitude of differences between proxy-reported and patient-reported outcomes did not vary much in the subgroup analysis.

**Conclusions:**

When there is a difference between proxy-reported and patient-reported outcomes, proxies tended to report more health and functional limitations among the elderly and disabled population. The extent of proxy response bias depended on the domain being tested and the nature of the question being asked. Researchers should accept proxy reports for sensory status and objective, observable, or easy questions. For physical, affective, cognitive, or social status and private, unobservable, or complex questions, proxy-reported outcomes should be used with caution when patient-reported outcomes are not available.

## Background

Proxies are individuals who answer questions for survey respondents. Ideally, survey respondents themselves are the best ones to answer the survey. In order to reduce non-response bias and make the survey representative of the study population, proxies are allowed for survey respondents who are not available (e.g., hospitalized or institutionalized) or unable (e.g., physical or cognitive impairments) to answer on their own behalf. Proxy responses are very common when surveys are conducted among the elderly or disabled population [1, 2]. In major Medicare surveys, proxy responses constituted 10 % to 30 % of all responses [3]. However, outcomes reported by proxies may be systematically different from those obtained from patients directly. Proxy response bias is the difference between the responses from proxy and survey respondents. The impact of proxy response bias on the validity of the estimates is a significant concern for researchers when surveys are conducted among the elderly or disabled.

Research has shown that health and functional status have direct impact on the demand for health care services and serve as key predictors of health care expenses [4, 5]. In order to better allocate scarce resources in health care, the accuracy of the estimation of health and functional status is of great importance. In the literature, however, there is a significant disagreement in some health and functional status measures reported by the patient and those reported by the proxy [6, 7]. In order to better utilize patient-reported health and functional status data, it is imperative to understand the potential proxy response bias in this important area.

Extensive literature has assessed the extent of proxy response bias. Much of this work has focused on controlling observed subject characteristics [3, 8, 9]. Since unobserved subject characteristics (mainly physical or cognitive impairments) are not identified, many of the existing studies are subject to omitted variable bias. Some estimates in published studies may be not valid and not give enough information about the extent of proxy response bias.

To date, literature has focused on the extent of proxy response bias among survey respondents who are able to provide responses but are not available at the time of the interview [3, 8]. There has been little attention given to survey respondents who cannot provide responses for themselves. Among the elderly, 28.5 % have physical disabilities and 9.5 % have cognitive disabilities; among the disabled, 53.6 % have physical disabilities and 37.8 % have cognitive disabilities [10]. Given the high proportion of the elderly and disabled who may be unable to respond, it is important to understand the extent of proxy response bias among these groups.

This study has two objectives: (1) to examine the presence, direction, and magnitude of possible differences between proxy-reported and patient-reported outcomes in health and functional status measures among Medicare beneficiaries surveyed in the Medicare Current Beneficiary Survey (MCBS); (2) to assess whether the extent of proxy response bias varies by the relationship between the subject and the proxy (spouse, non-spouse relative, and non-relative). We hypothesize that differences exist between proxy-reported and patient-reported outcomes in some domains of health and functional status and the extent of differences does not vary by the relationship between the subject and the proxy.

## Methods

### Data and study sample

We used data from the MCBS for this study. The MCBS is a longitudinal panel survey of Medicare beneficiaries, including community-dwelling and facility-dwelling beneficiaries, conducted by the Centers for Medicare and Medicaid Services (CMS). Survey respondents were interviewed 3 times a year over 4 years. It collects information about demographic and socioeconomic characteristics, health and functional status, service use, and health care spending for persons covered by Medicare. The MCBS is an appropriate dataset for this study in that it contains Medicare enrollment and claims data in addition to survey data. Because Medicare enrollment and claims data are independent to the survey, they are not subject to proxy response bias.

This study is a pooled cross-sectional study for a nationally representative sample of community-dwelling Medicare beneficiaries from 2006 to 2011. Because facility-dwelling beneficiaries do not have patient-reported data, we only included community-dwelling beneficiaries in the study.

### Measures

Survey respondents can respond either for themselves or via proxies. The best one to respond to the MCBS is the survey respondent. An effort is made to interview the survey respondent directly. In case the survey respondent is unable or not available to respond, he or she needs to name a proxy. In many cases, a spouse or child will serve as a proxy. But the proxy is not required to be a relative of the survey respondent. The outcome of interest in the study is health and functional status. It was assessed across five domains: physical, affective, cognitive, social, and sensory status. Health and functional status was measured by the percentage of limitations reported by survey respondents or proxies.

### Statistical analysis

The study used propensity score matching to balance the distribution of measured covariates between patient-report and proxy-report groups. Specifically, five steps were used to estimate the proxy response bias. In the first step, we used univariate relative risk regression to calculate unadjusted relative risk (RR) and 95 % confidence interval (CI) for each heath and functional limitation between patient-reports and proxy-reports. In step 2, we assessed the differences in socio-demographic characteristics and chronic conditions between two groups by using the chi-square test. Socio-demographic characteristics and chronic conditions may confound the association between types of responses and health and functional limitations. In step 3, we conducted a multivariate logistic regression. In the model, the dependent variable was the log of proxy and independent variables were a set of conditioning variables. Conditioning variables were restricted to those from Medicare enrollment and claims data; those variables included age, gender, race, education, marital status, household size, income, Medicare status, Charlson Comorbidity Index (CCI), and dementia. Based on the values of conditioning variables, each subject had an estimated propensity score, which is the predicted probability of using a proxy. In step 4, Greedy 5-to-1 digit matching was used to create matched samples [11]. Patient-reports were matched to proxy-reports in a 1:1 ratio (without replacement). With this matching method, patient-reports were first matched to proxy-reports with the same 5 digits. For those that did not match, patient-reports were matched to proxy-reports with the same 4 digits. Similar processes were continued until the remaining patient-reports were matched to the remaining proxy-reports with the same 1 digit. In the last step, matched patient-report and proxy-report samples were compared to assess the extent of proxy response bias. Conditional Poisson regression was used to analyze matched-pair data and calculate adjusted RR and 95 % CI for each health and functional limitation. Alternative matching techniques, including Kernel matching, radius matching with caliper 0.001, and Mahalanobis metric matching, were conducted as a sensitivity analysis to test the robustness of the results.

In Objective 2, we conducted a stratified analysis of Objective 1. The same matching technique was used in the stratified analysis. The only difference between the two aims was the number of comparisons being made. In Objective 2, proxy-reports were divided into three subgroups by the relationship between the subject and the proxy. Hence, we have three comparison groups: (1) spouse proxy-reports vs. patient-reports; (2) non-spouse relative proxy-reports vs. patient-reports; and (3) non-relative proxy-reports vs. patient-reports.

The study protocol was approved by the University of South Carolina Institutional Review Board. The study adhered to the Strengthening the Reporting of Observational Studies in Epidemiology (STROBE) checklists for cross-sectional studies. All analyses were performed using SAS Software version 9.4 (Statistical Analysis Systems, Cary, NC) and STATA version 13 (STATA Corp, College Station, TX).

## Results

The study identified a total of 76,115 person-years of patient-reports and 8,822 person-years of proxy-reports. Among proxy-reports, most of them were non-spouse relative proxy-reports (n = 5,126), followed by spouse (n = 3,011) and non-relative proxy-reports (n = 684).

The socio-demographic characteristics differed significantly between survey respondents who self-reported and those who are proxy-reported (Table 1). Survey respondents reported via proxies were more likely to be male, non-white, single, disabled, older than 85 years, less than a high school education, larger household size, and lower annual income. Patient-reports also differed significantly from proxy-reports in some self-reported chronic conditions. Especially, proxy-reports were associated with significantly more physical (measured by CCI) and cognitive impairments (measured by dementia). Distributions of some socio-demographic characteristics and chronic conditions were found to be uneven between patient-reports and proxy-reports.

**Table 1:** Characteristics of patient-reports and proxy-reports among medicare beneficiaries

	Unadjusted analysis			Adjusted analysis		
	Patient-report (n = 76,115)	Proxy-report (n = 8,822)	P	Patient-report (n = 7,780)	Proxy-report (n = 7,780)	P
	%	%		%	%	
Age			<0.0001			0.1582
<65	15.77	32.48		33.56	32.44	
65-74	36.89	19.64		21.16	20.78	
75-84	34.08	25.58		26.00	26.17	
85+	13.26	22.30		19.28	20.60	
Gender			<0.0001			0.0277
Male	44.06	58.38		60.06	58.33	
Female	55.94	41.62		39.94	41.67	
Race			<0.0001			0.1233
Non-Hispanic white	78.56	63.52		66.11	65.01	
Non-Hispanic black	9.54	14.14		13.70	13.33	
Hispanic	7.71	14.60		13.46	14.14	
Other	4.19	7.75		6.74	7.52	
Education			<0.0001			0.5539
Less than high school	24.10	45.40		44.33	43.66	
High school graduate	30.66	30.43		31.40	31.41	
Some college	23.36	13.54		13.95	13.92	
College graduate	21.88	10.63		10.32	11.00	
Marital status			<0.0001			0.9837
Married	50.13	42.28		44.02	43.89	
Widowed	28.68	24.70		23.48	23.59	
Single	21.19	33.01		32.49	32.52	
Household size			<0.0001			0.4089
One person	32.86	12.04		13.33	12.65	
Two people	50.46	49.39		50.40	51.12	
Three or more people	16.68	38.57		36.27	36.23	
Income			<0.0001			0.6225
<$25,000 per year	50.22	71.83		70.69	70.33	
≥ $25,000 per year	49.78	28.17		29.31	29.67	
Medicare status			<0.0001			0.0923
Aged	83.97	67.29		66.00	67.28	
Disabled	16.03	32.71		34.00	32.72	
CCI			0.0442			0.5235
0	76.62	76.13		76.88	76.62	
1	13.71	13.50		13.53	13.39	
2	6.62	6.79		6.54	6.52	
3+	3.05	3.58		3.05	3.47	
Dementia			<0.0001			0.4386
Yes	5.52	19.42		16.43	16.89	
No	94.48	80.58		83.57	83.11	

CCI: Charlson Comorbidity Index

Except for difficulties in stooping/crouching/kneeling, proxy-reports were associated with significantly higher percentages of health and functional limitations compared with patient-reports (Table 2). The magnitude of differences between two types of responses varied by domains and specific questions within domains. The observed differences can be attributed to non-random allocation or proxy response bias.

**Table 2:** Unadjusted and adjusted comparisons between patient-reports and proxy-reports in health and functional limitations

	Unadjusted analysis				Adjusted analysis			
	Patient-report (n = 76,115)	Proxy-report (n = 8,822)	RR (95 % CI)		Patient-report (n = 7,780)	Proxy-report (n = 7,780)	RR (95 % CI)	
	%	%			%	%		
Physical status								
ADL								
Bathing/showering	9.80	33.15	3.38 (3.26, 3.51)	*	13.60	31.61	2.32 (2.18, 2.47)	*
Dressing	6.45	26.32	4.08 (3.90, 4.26)	*	9.75	25.08	2.57 (2.38, 2.78)	*
Eating	2.16	11.36	5.25 (4.87, 5.66)	*	3.48	10.76	3.10 (2.71, 3.53)	*
Get in/out of bed/chair	13.22	26.30	1.99 (1.91, 2.07)	*	18.10	25.48	1.41 (1.33, 1.49)	*
Walking	26.69	39.79	1.49 (1.45, 1.53)	*	33.59	38.86	1.16 (1.11, 1.20)	*
Using the toilet	4.62	18.67	4.04 (3.83, 4.27)	*	6.48	17.67	2.73 (2.48, 3.00)	*
IADL								
Telephone	5.50	33.41	6.08 (5.83, 6.34)	*	8.66	32.00	3.70 (3.42, 3.99)	*
Doing light housework	12.58	37.91	3.01 (2.91, 3.12)	*	18.66	36.50	1.96 (1.85, 2.06)	*
Doing heavy housework	36.53	58.22	1.59 (1.56, 1.63)	*	44.31	57.00	1.28 (1.24, 1.32)	*
Preparing meals	9.38	44.91	4.79 (4.63, 4.95)	*	15.44	43.14	2.79 (2.63, 2.96)	*
Shopping	14.20	51.02	3.59 (3.50, 3.69)	*	21.28	49.38	2.32 (2.21, 2.43)	*
Managing money	6.73	55.39	8.24 (7.97, 8.51)	*	14.12	54.11	3.83 (3.62, 4.06)	*
Mobility								
Stooping/crouching/kneeling	72.52	71.72	0.99 (0.98, 1.00)		73.72	71.60	0.97 (0.95, 0.99)	*
Lifting/carrying 10 pounds	37.98	54.69	1.44 (1.41, 1.47)	*	45.70	53.90	1.18 (1.14, 1.22)	*
Extending arms above shoulder	27.73	41.75	1.51 (1.47, 1.55)	*	32.60	41.18	1.26 (1.21, 1.32)	*
Writing/handling object	26.82	41.14	1.53 (1.49, 1.58)	*	31.41	40.28	1.28 (1.23, 1.34)	*
Walking 1/4 mile or 2–3 blocks	48.88	62.42	1.28 (1.25, 1.30)	*	56.57	61.55	1.09 (1.06, 1.12)	*
Affective status								
Depressed	61.37	67.84	1.11 (1.09, 1.12)	*	65.76	67.87	1.03 (1.01, 1.05)	*
Losing interest	14.23	23.46	1.65 (1.58, 1.72)	*	20.79	23.31	1.12 (1.06, 1.19)	*
Cognitive status								
Memory loss	10.91	36.74	3.37 (3.25, 3.48)	*	18.09	35.52	1.96 (1.86, 2.07)	*
Decision making	7.10	40.65	5.72 (5.52, 5.94)	*	13.76	39.29	2.85 (2.69, 3.03)	*
Concentrating	15.18	46.45	3.06 (2.97, 3.15)	*	25.08	45.17	1.80 (1.72, 1.88)	*
Social status								
Social activities	35.14	54.20	1.54 (1.51, 1.58)	*	44.82	53.79	1.20 (1.16, 1.24)	*
Sensory status								
Seeing	31.22	35.40	1.13 (1.10, 1.17)	*	34.44	34.91	1.01 (0.97, 1.06)	
Hearing	36.83	39.23	1.07 (1.04, 1.10)	*	36.41	39.57	1.09 (1.05, 1.13)	*
Eating solid foods	14.87	21.32	1.43 (1.37, 1.50)	*	20.02	21.09	1.05 (0.99, 1.12)	

RR: Relative risk; CI: Confidence interval; ADL: Activities of daily living; IADL: Instrumental activities of daily living; *: Significant at 0.05 level

After applying the propensity score matching, we identified 7,780 person-years of patient-reports paired with 7,780 person-years of proxy-reports. Most characteristics were similar between two types of responses (Table 1). Proxy response bias was not observed in seeing (RR: 1.01, 95 % CI: 0.97-1.06) and eating solid foods (RR: 1.05, 95 % CI: 0.99-1.12) and was very small in hearing (RR: 1.09, 95 % CI: 1.05-1.13) (Table 2). Four other domains were found to have proxy response bias even after propensity score matching. Proxies tended to report more health and functional limitations in comparison to survey respondents themselves. Two domains had small proxy response biases: affective (RR: 1.03-1.12) and social status (RR: 1.20). The cognitive status domain (RR: 1.80-2.85) had moderate proxy response bias. Within the physical status domain, small proxy response bias was found in mobility (RR: 0.97-1.28) and moderate to large proxy response biases were found in activities of daily living (ADL) (RR: 1.16-3.10) and instrumental activities of daily living (IADL) (RR: 1.28-3.83). Significant items and proxy response bias in each domain were summarized in Table 3. A question regarding survey respondents’ difficulties in managing money was associated with the largest proxy response bias (RR = 3.83). The results were robust in the sensitivity analysis (Table 4).

**Table 3:** Summary of significant items and proxy response bias in each domain

	Items		Proxy response bias		
	Total	Significant	Presence	Direction	Magnitude
Physical status					
ADL	6	6	Yes	Positive	1.16-3.10
IADL	6	6	Yes	Positive	1.28-3.83
Mobility	5	5	Yes	Positive	0.97-1.28
Affective status	2	2	Yes	Positive	1.03-1.12
Cognitive status	3	3	Yes	Positive	1.80-2.85
Social status	1	1	Yes	Positive	1.20
Sensory status	3	1	No	-	-

ADL: Activities of daily living; IADL: Instrumental activities of daily living

**Table 4:** Sensitivity analysis to compare Greedy 5-to-1 matching and other propensity score matching techniques

	Greedy 5 to 1 digit matching		Kernel matching		Radius matching		Mahalanobis metric matching	
	RR		RR		RR		RR	
	(95 % CI)		(95 % CI)		(95 % CI)		(95 % CI)	
Physical status								
ADL								
Bathing/showering	2.32 (2.18, 2.47)	*	2.34 (2.18, 2.51)	*	2.33 (2.17, 2.50)	*	2.21 (2.06, 2.36)	*
Dressing	2.57 (2.38, 2.78)	*	2.66 (2.46, 2.89)	*	2.62 (2.41, 2.84)	*	2.69 (2.48, 2.91)	*
Eating	3.10 (2.71, 3.53)	*	3.31 (2.90, 3.79)	*	3.20 (2.80, 3.66)	*	3.91 (3.39, 4.50)	*
Get in/out of bed/chair	1.41 (1.33, 1.49)	*	1.44 (1.35, 1.54)	*	1.41 (1.32, 1.51)	*	1.62 (1.52, 1.74)	*
Walking	1.16 (1.11, 1.20)	*	1.17 (1.12, 1.24)	*	1.16 (1.11, 1.22)	*	1.20 (1.14, 1.27)	*
Using the toilet	2.73 (2.48, 3.00)	*	2.84 (2.57, 3.13)	*	2.84 (2.57, 3.13)	*	2.62 (2.38, 2.88)	*
IADL								
Telephone	3.70 (3.42, 3.99)	*	4.08 (3.74, 4.44)	*	4.03 (3.70, 4.38)	*	3.37 (3.11, 3.64)	*
Doing light housework	1.96 (1.85, 2.06)	*	1.98 (1.86, 2.10)	*	1.97 (1.85, 2.10)	*	2.01 (1.89, 2.15)	*
Doing heavy housework	1.28 (1.24, 1.32)	*	1.28 (1.23, 1.34)	*	1.28 (1.22, 1.34)	*	1.30 (1.24, 1.36)	*
Preparing meals	2.79 (2.63, 2.96)	*	2.88 (2.70, 3.08)	*	2.84 (2.66, 3.03)	*	2.74 (2.57, 2.93)	*
Shopping	2.32 (2.21, 2.43)	*	2.39 (2.26, 2.53)	*	2.34 (2.21, 2.48)	*	2.06 (1.95, 2.18)	*
Managing money	3.83 (3.62, 4.06)	*	4.23 (3.95, 4.53)	*	4.01 (3.75, 4.28)	*	3.40 (3.19, 3.62)	*
Mobility								
Stooping/crouching/kneeling	0.97 (0.95, 0.99)	*	0.96 (0.93, 1.00)		0.97 (0.94, 1.01)		1.01 (0.97, 1.05)	
Lifting/carrying 10 pounds	1.18 (1.14, 1.22)	*	1.19 (1.14, 1.24)	*	1.18 (1.13, 1.23)	*	1.27 (1.22, 1.33)	*
Extending arms above shoulder	1.26 (1.21, 1.32)	*	1.25 (1.19, 1.32)	*	1.24 (1.18, 1.31)	*	1.26 (1.20, 1.33)	*
Writing/handling object	1.28 (1.23, 1.34)	*	1.29 (1.23, 1.36)	*	1.29 (1.22, 1.35)	*	1.35 (1.29, 1.43)	*
Walking 1/4 mile or 2–3 blocks	1.09 (1.06, 1.12)	*	1.09 (1.04, 1.13)	*	1.08 (1.04, 1.12)	*	1.12 (1.08, 1.17)	*
Affective status								
Depressed	1.03 (1.01, 1.05)	*	1.03 (0.99, 1.07)		1.03 (0.99, 1.07)		1.06 (1.02, 1.10)	*
Losing interest	1.12 (1.06, 1.19)	*	1.16 (1.09, 1.24)	*	1.14 (1.07, 1.22)	*	1.21 (1.13, 1.30)	*
Cognitive status								
Memory loss	1.96 (1.86, 2.07)	*	2.00 (1.88, 2.12)	*	1.98 (1.86, 2.10)	*	1.80 (1.69, 1.91)	*
Decision making	2.85 (2.69, 3.03)	*	3.10 (2.89, 3.32)	*	2.99 (2.79, 3.20)	*	2.64 (2.48, 2.82)	*
Concentrating	1.80 (1.72, 1.88)	*	1.95 (1.85, 2.06)	*	1.90 (1.80, 2.00)	*	1.74 (1.65, 1.84)	*
Social status								
Social activities	1.20 (1.16, 1.24)	*	1.20 (1.15, 1.25)	*	1.19 (1.14, 1.24)	*	1.19 (1.14, 1.24)	*
Sensory status								
Seeing	1.01 (0.97, 1.06)		1.01 (0.96, 1.07)		1.01 (0.96, 1.06)		1.06 (1.01, 1.12)	*
Hearing	1.09 (1.05, 1.13)	*	1.09 (1.03, 1.14)	*	1.10 (1.05, 1.16)	*	1.10 (1.05, 1.16)	*
Eating solid foods	1.05 (0.99, 1.12)		1.07 (1.00, 1.15)		1.07 (1.00, 1.15)		1.15 (1.08, 1.24)	*

RR: Relative risk; CI: Confidence interval; ADL: Activities of daily living; IADL: Instrumental activities of daily living *: Significant at 0.05 level

Characteristics were balanced between two types of responses in the stratified analysis. (Data not shown) With few exceptions, the presence, direction, and magnitude of differences between proxy-reported and patient-reported outcomes did not vary much in the subgroup analysis (Table 5). That is to say, the ways spouse, non-spouse relative, and non-relative proxies respond to the survey were very similar.

**Table 5:** Subgroup analysis of comparisons between patient-reports and proxy-reports in health and functional limitations

	Patient-report (n = 2,775)	Spouse proxy-reports (n = 2,775)	Adjusted RR (95 % CI)		Patient-report (n = 4,434)	Non-spouse Relative proxy-reports (n = 4,434)	Adjusted RR (95 % CI)		Patient-report (n = 570)	Non-relative proxy-reports (n = 570)	Adjusted RR (95 % CI)	
	%	%			%	%			%	%		
Physical status												
ADL												
Bathing/showering	8.90	23.34	2.62 (2.30, 2.99)	*	16.56	36.38	2.20 (2.04, 2.37)	*	13.56	34.92	2.58 (2.05, 3.24)	*
Dressing	7.68	20.36	2.65 (2.29, 3.07)	*	11.17	27.73	2.48 (2.26, 2.73)	*	8.79	27.51	3.13 (2.34, 4.19)	*
Eating	2.78	9.02	3.25 (2.54, 4.17)	*	3.98	11.78	2.96 (2.52, 3.49)	*	2.99	11.29	3.78 (2.27, 6.28)	*
Get in/out of bed/chair	13.84	23.44	1.69 (1.52, 1.89)	*	20.86	26.55	1.27 (1.18, 1.37)	*	17.40	27.16	1.56 (1.25, 1.95)	*
Walking	26.64	35.99	1.35 (1.25, 1.46)	*	38.11	40.50	1.06 (1.01, 1.12)	*	32.34	40.21	1.24 (1.07, 1.45)	*
Using the toilet	4.79	14.36	2.99 (2.48, 3.61)	*	7.48	19.38	2.59 (2.30, 2.92)	*	6.87	20.46	2.98 (2.13, 4.17)	*
IADL												
Telephone	9.21	25.58	2.78 (2.44, 3.16)	*	8.64	36.31	4.20 (3.80, 4.66)	*	5.99	30.05	5.03 (3.57, 7.07)	*
Doing light housework	12.51	29.95	2.39 (2.14, 2.68)	*	22.48	40.14	1.79 (1.67, 1.91)	*	18.57	38.18	2.05 (1.69, 2.49)	*
Doing heavy housework	30.88	46.76	1.51 (1.41, 1.62)	*	52.40	63.15	1.20 (1.16, 1.25)	*	45.86	57.48	1.25 (1.12, 1.40)	*
Preparing meals	9.69	31.43	3.23 (2.85, 3.67)	*	19.26	49.15	2.55 (2.38, 2.73)	*	12.80	49.43	3.86 (3.06, 4.87)	*
Shopping	13.20	35.00	2.66 (2.40, 2.95)	*	26.40	57.40	2.17 (2.06, 2.30)	*	20.92	55.25	2.64 (2.23, 3.13)	*
Managing money	7.55	32.96	4.36 (3.80, 4.99)	*	17.96	65.43	3.64 (3.41, 3.89)	*	15.71	65.19	4.14 (3.41, 5.04)	*
Mobility												
Stooping/crouching/kneeling	71.15	74.90	1.05 (1.02, 1.09)	*	75.45	69.85	0.93 (0.90, 0.95)	*	72.76	69.16	0.95 (0.88, 1.03)	
Lifting/carrying 10 pounds	32.05	47.46	1.48 (1.39, 1.58)	*	53.82	57.84	1.07 (1.04, 1.11)	*	49.12	54.72	1.11 (1.00, 1.24)	
Extending arms above shoulder	26.18	41.03	1.57 (1.45, 1.69)	*	36.24	41.62	1.15 (1.09, 1.21)	*	35.50	38.54	1.09 (0.94, 1.26)	
Writing/handling object	26.82	38.25	1.43 (1.32, 1.54)	*	34.12	41.80	1.23 (1.16, 1.29)	*	32.51	38.37	1.18 (1.01, 1.38)	*
Walking 1/4 mile or 2–3 blocks	47.37	60.57	1.28 (1.22, 1.34)	*	62.15	62.64	1.01 (0.98, 1.04)		58.00	57.95	1.00 (0.91, 1.10)	
Affective status												
Depressed	54.12	61.78	1.14 (1.09, 1.19)	*	72.09	71.12	0.99 (0.96, 1.01)		73.24	72.74	0.99 (0.92, 1.07)	
Losing interest	12.36	21.66	1.75 (1.56, 1.97)	*	25.19	24.45	0.97 (0.90, 1.04)		27.82	22.63	0.81 (0.66, 1.00)	
Cognitive status												
Memory loss	12.46	30.53	2.45 (2.20, 2.73)	*	21.23	38.76	1.83 (1.71, 1.95)	*	21.13	34.94	1.65 (1.36, 2.01)	*
Decision making	8.49	27.81	3.27 (2.88, 3.72)	*	16.63	45.63	2.74 (2.55, 2.95)	*	17.22	46.44	2.70 (2.21, 3.28)	*
Concentrating	16.69	32.84	1.97 (1.80, 2.16)	*	29.72	51.90	1.75 (1.66, 1.84)	*	29.93	53.48	1.79 (1.55, 2.06)	*
Social status												
Social activities	33.25	50.00	1.50 (1.41, 1.60)	*	51.21	56.12	1.10 (1.05, 1.14)	*	51.41	54.30	1.06 (0.94, 1.19)	
Sensory status												
Seeing	30.84	33.27	1.08 (1.00, 1.16)		36.41	36.28	1.00 (0.94, 1.05)		36.57	32.38	0.89 (0.76, 1.03)	
Hearing	43.64	49.73	1.14 (1.08, 1.20)	*	32.30	34.84	1.08 (1.02, 1.14)	*	33.04	26.52	0.81 (0.68, 0.95)	*
Eating solid foods	14.82	19.23	1.30 (1.16, 1.45)	*	22.99	22.27	0.97 (0.90, 1.05)		22.11	20.91	0.95 (0.75, 1.19)	

RR: Relative risk; CI: Confidence interval; ADL: Activities of daily living; IADL: Instrumental activities of daily living; *: Significant at 0.05 level

## Discussion

The study successfully controlled for major confounding variables of proxy response bias. The distributions of socio-demographic characteristics and chronic conditions between two types of responses were not even in the study. The presence of proxy response bias observed in the unadjusted analysis might be attributed to socio-demographic characteristics and chronic conditions differences. For example, survey respondents older than 85 years were more likely to respond to the survey via proxies. They were also more likely to have health and functional limitations. Another example was dementia patients. They were more likely to use proxies to respond and have more health and functional limitations. Existing studies also found that socio-demographic characteristics confounded the association between proxy response and health and functional limitations [3, 8, 9]. So unevenly distributed socio-demographic characteristics and chronic conditions might serve as confounding variables in the study. These major confounders were controlled using the propensity score matching approach.

Given the high base rate of health and functional limitations among the elderly and disabled, it seems proxies are more likely to assume that an elderly or disabled survey respondent has a limitation unless they have sufficient information about the limitation. Under this assumption, proxies tended to report more health and functional limitations for elderly or disabled survey respondents.

The extent of proxy response bias depended on the domain being tested. Survey respondents with sensory limitations can be easily observed by proxies. However, physical, affective, cognitive, or social limitations are sometimes hard to observe. So the sensory status domain was less likely to suffer proxy response bias.

The nature of the question being asked can also impact the extent of proxy response bias. Proxies are good reporters for objective, observable, or easy questions but usually do not have enough information on private, unobservable, or complex questions. For example, a question regarding survey respondents’ difficulties in walking 1/4 mile or 2–3 blocks is objective, observable to proxies, and easy to answer. Even though proxies still report more limitations, the magnitude of proxy response bias was very small. On the contrary, difficulty in managing money is very complex. As a result, large proxy response bias was observed for this question. Another example is a question regarding survey respondents’ difficulties in using the toilet. This question is about an activity that is private and unobservable to proxies. So we observed large proxy response bias for this question.

The MCBS is a widely used dataset in conducting health services research among Medicare beneficiaries. The issue of non-response bias in the MCBS was previously investigated [12]. The study found that the MCBS was not subject to non-response bias. The current study is the first to investigate proxy response bias in the MCBS. When using proxy-reported data in the MCBS, most of the existing studies assumed that responses from survey respondents and proxies can be used interchangeable. According to the findings of this study, however, such an assumption is invalid. In order to improve use the MCBS, researchers should be aware of proxy response bias.

The study has limitations. First, even though we included some unobservable variables identified in previous studies, it is highly possible that we did not include other unobservable confounding variables. If that is the case, we were not able to simulate random allocation of all confounding variables. As a result, the study may be subject to omitted variable bias and will lead to type I error. Secondly, this study only investigated proxy response bias. The extent of self-report bias is unknown. Thirdly, this study matched patient-reports to proxy reports in a 1:1 ratio. Using 1:n matching could lead to higher bias, although it might increase estimate precision [13].

The study has the following four strengths. First, survey respondents who cannot respond for themselves were included in the analysis. Secondly, cognitive impairments were included in the group of conditioning variables. According to the literature, cognitive impairments are the major reason for non-random allocation of survey respondents between proxy-reports and patient-reports. Thirdly, all the conditioning variables are independent to the survey. As a result, they are free from proxy response bias. Finally, in addition to Greedy 5 to 1 digit matching, this study used alternative matching techniques as a sensitivity analysis [14–16]. The presence, direction, and magnitude of proxy response bias are similar to Greedy 5 to 1 matching used by the study. Therefore, the results are robust to alternative matching techniques.

## Conclusions

Proxy response bias was present in the physical, affective, cognitive, and social status domains but not in the sensory status domain. Specifically, proxies tended to report more health and functional limitations among the elderly or disabled population compared with patient-reports. The magnitude of proxy response bias was large in questions involving private information, unobservable factors, or complex answers. When assessing the impact of different relationships on proxy response bias, the presence, direction, and magnitude all remained the same. When patient-reported outcomes are not available, researchers should accept proxy reports for sensory status and objective, observable, or easy questions. For physical, affective, cognitive, or social status and private, unobservable, or complex questions, proxy reports should be used with caution when patient-reported outcomes are not available.

The current study provides useful findings for survey organizations that wish to minimize proxy response bias. At the questionnaire development stage, objective, observable, or easy questions that do not call for judgments by proxies are preferred. At the survey execution stage, when the subject is unable to respond, interviewers should identify a proxy who is familiar with the questions being asked. The results of this study will also help researchers better use survey data. When using survey data obtained from proxies, researchers should describe possible effects of proxy response bias on study results.

## Abbreviations

ADL: activities of daily living
CCI: Charlson Comorbidity Index
CI: confidence interval
CMS: Centers for Medicare and Medicaid Services
IADL: instrumental activities of daily living
MCBS: Medicare Current Beneficiary Survey
RR: relative risk
